# Conceptualising community engagement as an infinite game implemented through finite games of ‘research’, ‘community organising’ and ‘knowledge mobilisation’

**DOI:** 10.1111/hex.13801

**Published:** 2023-06-23

**Authors:** Tanvir C. Turin, Mashrur Kazi, Nahid Rumana, Mohammad A. A. Lasker, Nashit Chowdhury

**Affiliations:** ^1^ Department of Family Medicine, Department of Community Health Sciences, Cumming School of Medicine, O'Brien Institute for Public Health, Libin Cardiovascular Institute University of Calgary Calgary Alberta Canada; ^2^ Department of Family Medicine, Cumming School of Medicine University of Calgary Calgary Alberta Canada; ^3^ Community Scholar and Citizen Researcher Calgary Alberta Canada; ^4^ Community Champion and Citizen Researcher Calgary Alberta Canada

**Keywords:** civic engagement, community engagement, public involvement

## Abstract

Meaningful community engagement process involves focusing on the community needs, building community capacity and employing culturally tailored and community‐specific strategies. In the current practices of community‐engaged health and wellness research, generally, community engagement activities commence with the beginning of a particular research project on a specific topic and end with the completion of the project. The outcomes of the community engagement, including the trust, partnership and contribution of the community to research, thus remain limited to that specific project and are not generally transferred and fostered further to the following project on a different topic. In this viewpoint article, we discussed a philosophical approach to community engagement that proposes to juxtapose community engagement for the specific short‐term research project and the overarching long‐term programme of research with the finite game and infinite game concepts, respectively. A finite game is a concept of a game where the players are known, rules are fixed and when the agreed‐upon goal is achieved, the game ends. On the other hand, in infinite games, the players may be both known and unknown, have no externally fixed rules and have the objective of continuing the game beyond a particular research project. We believe community engagement needs to be conducted as an infinite game that is, at the programme of research level, where the goal of the respective activities is not to complete a research project but to successfully engage the community itself is the goal. While conducting various research projects, that is, finite games, the researchers need to keep an infinite game mindset throughout, which includes working with the community for a just cause, building trust and community capacity to maximise their contribution to research, prioritising community needs and having the courage to lead the community if need be.

**Patient or Public Contribution:** While preparing this manuscript, we have partnered actively with community champions, activists, community scholars and citizen researchers at the community level from the very beginning. We had regular interactions with them to get their valuable and insightful inputs in shaping our reflections. Their involvement as coauthors in this paper also provided a learning opportunity for them and facilitated them to gain insight on knowledge engagement. All authors support greater community/citizen/public involvement in research in an equitable manner.

## 1 INTRODUCTION

Community engagement is an umbrella term that encompasses a wide range of definitions. However, in simplistic terminology, community engagement seeks to collaborate with the community in an equitable and empowering manner to achieve sustainable outcomes.^1^ There is a multitude of benefits when it comes to community engagement. This process empowers the community to participate in the decision‐making process around different aspects of research, directly contributing to the concern at hand from their lived experience and relevant expertise. This promotes personal agency, well‐being and self‐confidence for the members of the community and opens up opportunities to act as community enablers for public health, injustice or indeed any other concern that is of importance to the community.^2^, ^3^ The fundamental approach to community engagement is to allow the fostering of impactful relationships between different stakeholders including with the community to where they belong. As community researchers, we encountered challenges when attempting to configure community engagement solely within the confines of a single research project. We found that our efforts to foster desired engagement and relationship building were falling short. However, once we shifted our perspective and began conceptualising community engagement as an integral part of a broader programme of research encompassing multiple projects, we started to experience greater success in terms of community trust, buy‐in and involvement. In this viewpoint article, we discussed a logical approach to community engagement that proposes juxtaposing community engagement for specific short‐term research projects and overarching long‐term programmes of research.

## 2 PRINCIPLES OF COMMUNITY ENGAGEMENT

The engagement process with the community is a continuous and long‐term endeavour. Atlee et al.^4^ identified seven principles as a way to govern community engagement. ‘Openness and Learning’ allows stakeholders to engage with the community by actively listening to their concerns, feedback and ideas. The principle of ‘Careful Planning and Preparation’ embodies the concept of creating a model of community engagement that is inclusive and has a clearly defined shared purpose. Actively involving members of the diverse community is another principle that Atlee et al.^4^ termed ‘Inclusion and Demographic Diversity’. Community engagement by definition is a collaborative process, however, the principle—‘Collaboration and Shared Purpose’ ensures that the collaboration is built on a purpose that interests and benefits both researchers and the communities. This is also key to the sustainability of the engagement; which was further reinforced by the ‘Sustained Engagement and Participatory Culture’ principle. Transparency is a crucial element between partners to establish a long, enriching and trusting relationship between the organisations, researchers and participants of the community—coined the ‘Transparency and Trust’ principle. Finally, the ‘Impact and Action’ principle underscores the importance of translating community engagement and the associated research into action to create real change and have an impact on the community in a positive manner.

## 3 COMMUNITY ENGAGEMENT THROUGH A STRATEGIC APPROACH

There are a number of community engagement strategies proposed and applied through various studies.^2^, ^5^ For meaningful community engagement, it has been universally emphasised to focus on developing equitable partnerships with the community, taking a community‐centred approach and employing culturally sensitive and community‐specific strategies.^6^ In health and wellness research, which is commonly driven by a time‐bound and target‐focused culture,^7^ developing a meaningful and long‐term research partnership with the community through continuous community engagement is often not stressed and therefore overlooked. This drives the researchers to focus on specific project‐based community member involvement strategies to have community representation in the projects. The participation ends with the duration of the research project.^8^ The success of the reach of community engagement remains limited to those participants who are probably only interested in a particular topic, incentives or other personal/professional gain as opposed to developing a genuine interest in engaging in the research. In addition, the funding and resources for community engagement are also mostly limited to a specific research project, thereby restraining researchers and the community from continuing the engagement beyond the project. Therefore, a strategy for community engagement needs to be tailored to the programme of research. A programme of research encompasses multiple interlinked research projects that cover a wide range of issues and concerns of the involved community. This approach enables the researchers and the community in question to carry out the achieved community engagement, outreach and outcomes over another related project.

## 4 TOWARD A COMMUNITY‐ENGAGED PROGRAMME OF RESEARCH

In the context of research, community engagement can be seen as a fundamental step toward community‐based participatory research or community‐engaged research (CEnR) because it builds the basis of a mutual partnership between researchers, organisations and members of the community.^5^, ^9^, ^10^, ^11^ This process provides a window for researchers to truly understand the community context and ecosystem, leading to research that is relevant and appropriate to the community. Researchers are able to strive toward an approach that best serves the needs and wants of the community and inspires citizen research, thus empowering members of the community in a collaborative manner. In this way, participants can shift the power dynamics in the researcher–participant relationship from observed to engaged.

We conducted a number of research projects on equitable access to care,^12^, ^13^, ^14^, ^15^ community health and wellness,^16^, ^17^ as well as job market integration and resettlement issues^18^, ^19^, ^20^ as part of our CEnR programme for immigrant/ethnic–minority communities in Canada. This research examined the barriers to healthcare access and unmet healthcare needs encountered by Bangladeshi Canadians.^12^, ^13^ Through our community conversations,^14^ we investigated potential solutions to these barriers and challenges that the community struggles with when accessing care. We also sought community input for issue prioritisation^15^ which guided our research approach and strategies and led us to focus on health literacy.^21^ We interacted with a variety of community groups and organisations during these studies and recognised that each community group and organisation has its own viewpoints, expectations, advantages and constraints. We developed plans for the purposeful and active participation of community members and organisations serving immigrant/ethnic‐minority groups in research, priority‐setting, cocreation of knowledge products and knowledge translation or mobilisation activities.

As our work progressed, we realised that we need to conceptualise community engagement at the programme level, not at the project level. Community engagement needs to be strategized as an approach to doing things inside the community and to explain the nature of our work to a wide range of the public and/or community members. It should not be conceptualised at a single research project level, because a single project level approach might lead to a parachute in and out scenario rather than maintaining a consistent presence. Our research programme‐level community engagement efforts paid off by achieving the participation of members of the community in our research projects (both problem identification and solution development). Our community engagement efforts contributed to building interpersonal trust and led to active collaboration across all the steps of the research process of brainstorming, planning, executing and disseminating results.

## 5 INFINITE AND FINITE GAME

For a better comprehension of our community engagement strategy, we draw on the concept of ‘infinite and finite games’.^22^ According to this concept, there are two types of games we engage in that are applicable in many aspects of our lives. These include education and career goals, work, business and essentially any social situation and activity where there are multiple participants and social, individual and systemic factors involved. Individuals need to follow certain rules, make decisions based on interactions with other individuals and consider multiple factors while striving to obtain certain outcomes. Finite games are set by specific objectives, timeframes, rules and boundaries. Finite players can either win or lose in this type of game.^22^, ^23^ On the other hand, infinite games are continuous activities without any designated beginning or end. In infinite games, the players are always learning and growing to advance a cause through building trusting teams while experiencing flexible growth.^22^, ^23^ The objective of the players in finite games is to win, and the winning or losing ends the game. In infinite games, the objective is not winning but rather ensuring the continuation of play, thus the game never ends. Similar to this concept, our community engagement efforts are centred around building trusting and collaborative relationships with communities.^22^, ^23^ An infinite player is motivated to keep the game going for as long as possible rather than looking for any immediate ‘win’.^23^ As such, community engagement needs to be seen as an infinite game and therefore needs to be played with an infinite mindset. When infinite games are approached by using a finite mindset, the outcomes lead to decreased participation, trust, innovation and engagement which is not the goal of community engagement efforts. This, ultimately, leads to increased frustration from both the research and community sides.

## 6 FACTORS SHAPING AN INFINITE MINDSET FOR COMMUNITY ENGAGEMENT

**Figure 1:**
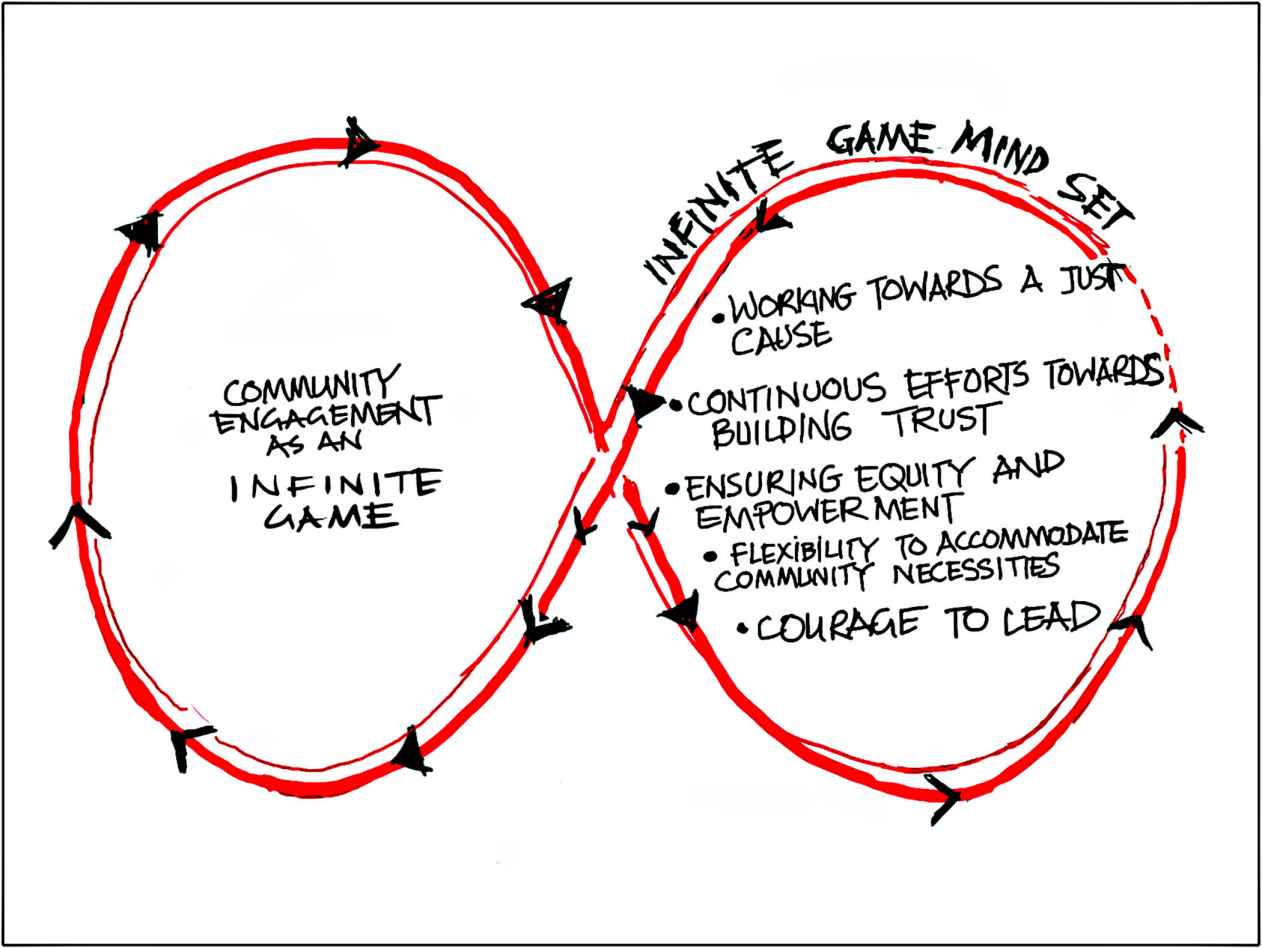
Infinite game mindset for community engagement.

As mentioned earlier, we need to conceptualise community engagement at the programme level, not at the project level. When meaningful community engagement for a programme of research is achieved, specific research projects requiring active participation of the community subsequently follow. Hence, we need to approach community engagement through an infinite game mindset,^24^ as illustrated in Figure 1.

Community engagement toward a just cause, such as ensuring equity for immigrant/ethnic‐minority communities, needs an infinite mindset. Community engagement thrives where there is a vision worth pursuing wholeheartedly when the vision is bigger than the researchers or the research projects. To advance the cause, the community needs to be on the side of the vision. This can be achieved through creating a relationship built on mutual trust, nonjudgement and shared benefit. The commitment to ensuring equitable and empowered involvement of the community in the process is another element that we always need to strive for. Without this approach, community engagement fails to mobilise the mass of people in the community. An infinite game approach also needs to ensure flexibility to accommodate the community's needs. This may warrant a change in the research focus based on experiential learning gained through community engagement. It calls for the fluidity of the researchers to be guided by the community regarding the focus of the programme of research. This approach ensures the community's long‐term engagement and contributes to long‐lasting trust. The courage to lead community engagement in these ways allows for innovation and the creation of new avenues of opportunity, thus perpetuating the infinite game.

## 7 SERIES OF FINITE GAMES CONTRIBUTING TO THE OVERARCHING INFINITE GAME

**Figure 2:**
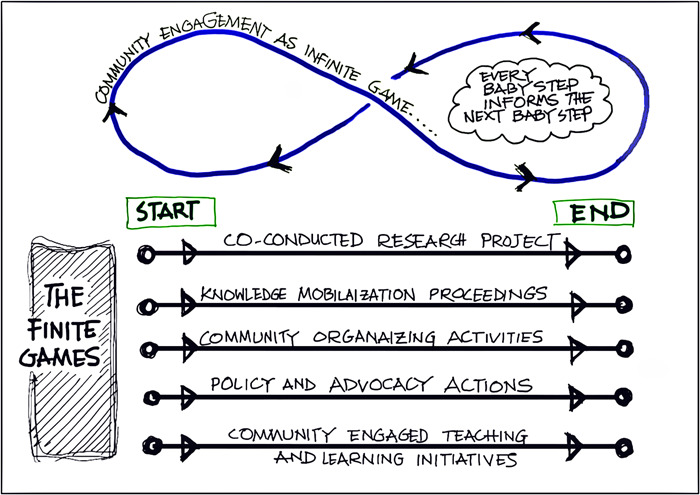
Finite games within the infinite game of community engagement.

An infinite game does not exclude finite games. Rather, an infinite game is a context within which we can have a series of finite games (Figure 2). Finite games may exist within the infinite game, acting as checkpoints along the journey.^24^ However, these games should not dictate the endpoint of the journey and should not derail the focus of the main mission. For example, within the infinite game of keeping up the health of community members, we can employ finite games such as playing indoor soccer in winter, outdoor cricket in summer or mall walking during bad weather days.

We conceptualised that our individual research projects can be considered as a finite game. A research project has a start and an end with a number of possible tangible outputs. Furthermore, dissemination activities can be planned to take the findings and knowledge to the community. Also, we approached our research projects for not only knowledge creation but also to employ as a tool for engagement. Moreover, within our infinite game of community engagement, our finite game research projects have been acting as our milestones through completing a manuscript or conducting dissemination sessions. Table 1 shows the concurrence of the concept of infinite and finite games with respect to our programme of research.

**Table 1:** The juxtaposition of the concept of infinite and finite games with respect to our programme of research.

The infinite game	The finite games	Example of finite games
Community engagement with racialized/ethnic‐minority communities for community health and wellness.	Co‐conducted research projects.	Equitable access to primary care.
		Health and wellness literacy and determinants in the community.
		Mental wellness needs of nonhealth essential workers.
		Addressing digital inequity and the digital divide
		Job market integration of internationally trained health professionals.
		Community‐based knowledge engagement hub.
	Knowledge dissemination activities.	Preparing and copresenting research results.
		Cowriting and publishing manuscripts.
		Writing articles for ethnic media.
		Arranging health and wellness workshops.
		Teaching and mentoring different levels of learners.
	Community organising actions.	Youth summer programme.
		Community scholar and citizen researcher programme.
		Newcomer research network.
		Alternative career mentoring.
		Coaching for professional development.
		Community Advisory Board or Community Advisory Group for research projects.
	Policy and advocacy.	Codeveloping and disseminating policy briefs, reports, white papers, or concept notes.
		Coadvocating for the cause through insider ownership and/or through outsider championship, as appropriate.
		Contributing to provincial and national level working groups.

## 8 CONCLUSION

The article refers to the principles and importance of community engagement in research. Drawing on the experience with multiple communities during our programme of research, we point out the need for strategic change in the community engagement process. Specifically, we refer to the concept of infinite and finite games in our community engagement strategy. We juxtapose short‐term specific project‐focused community engagement with the finite games where the players (e.g., smaller community subgroup and a few stakeholders of the project) are known, rules are fixed (e.g., community help in the recruitment or share lived experience and researchers design and lead the process, required performance measurement metrics to report, etc.), and when the agreed upon goal (e.g., completion of a research project or intervention) is achieved, the game ends. We advocate for a community engagement strategy for a programme of research instead of limiting it to one project. We do this by drawing on the infinite game concept where the players may be both known and unknown (e.g., research partners/collaborators, political/civil personalities, etc.), have no externally fixed rules (e.g., the researchers and community engage each other in multiple ways and beyond the methodological element of a study), and have the objective of continuing the game (i.e., the engagement) beyond a particular research project. The essence of this approach lies not in simply accomplishing a research project or paper, but rather in persistently pursuing research, community organising and knowledge mobilisation efforts that endure and contribute to the cause over time. It emphasises the ongoing commitment to community in a sustainable manner, beyond mere project completions. This transformative switch enhances the application and implementation of the principles of community engagement, thereby perpetuating the benefits to the community.

## AUTHOR CONTRIBUTIONS

Tanvir C. Turin, Nahid Rumana and Mohammad A. A. Lasker conceived the paper. Tanvir C. Turin, Mashrur Kazi and Nashit Chowdhury drafted the paper. All authors provided critical input for multiple drafts. Nahid Rumana and Mohammad A. A. Lasker critically reviewed the manuscript. Nahid Rumana and Mohammad A. A. Lasker provided important perspectives as community member researchers. Tanvir C. Turin acts as guarantor to this article.

## ACKNOWLEDGEMENTS

We acknowledge Mr. Tanzir Chowdhury Tuhin's contributions to drawing the figures used in this manuscript.

## CONFLICT OF INTEREST STATEMENT

The authors declare no conflict of interest.

## Data Availability

This is a viewpoint article, thus there is no data viable with this article.
